# Cerebral perfusion in untreated, controlled, and uncontrolled hypertension

**DOI:** 10.1177/0271678X221124644

**Published:** 2022-09-13

**Authors:** Isabel N Christie, Rowan Windsor, Henk JMM Mutsaerts, Therese Tillin, Carole H Sudre, Alun D Hughes, Xavier Golay, Alexander V Gourine, Patrick S Hosford

**Affiliations:** 1 Centre for Cardiovascular and Metabolic Neuroscience, Neuroscience, Physiology and Pharmacology, University College London, London, UK; 2 Department of Radiology and Nuclear Medicine, Amsterdam University Medical Center, Amsterdam, The Netherlands; 3 MRC Unit for Lifelong Health & Ageing, Population Science & Experimental Medicine, Faculty of Population Health Sciences, University College London, London, UK; 4 Institute of Cardiovascular Science, Population Science & Experimental Medicine, Faculty of Population Health Sciences, University College London, London, UK; 5 Queen Square Institute of Neurology, University College London, London, UK

**Keywords:** Cerebral blood flow, brain, hypertension, MRI, perfusion

## Abstract

This study evaluated the association between systemic arterial blood pressure and cerebral perfusion in 740 participants of the UK's largest tri-ethnic study with measurements of cerebral blood flow (CBF) performed using arterial spin labelling MRI. A significant negative correlation between blood pressure, age and CBF was observed across the patient cohort. The lowest CBF values were recorded in the group of patients with hypertension that were prescribed with anti-hypertensive drugs, but uncontrolled on medication. These findings confirm that hypertension is associated with reduced cerebral perfusion and highlight the importance of blood pressure control for the benefit of maintaining brain blood flow.

Hypertension affects 1.5 billion people worldwide and its prevalence increases as the global population ages. Hypertension is now considered one of the leading causes of age-related cognitive impairment and is strongly associated with reduced cerebral blood flow (CBF).^1,2^ There is significant evidence that reduced brain perfusion, sustained over many years, leads to progressive cognitive decline, development of dementia and neurodegenerative disease, such as Alzheimer’s disease.^1^ The effect of anti-hypertensive drugs on CBF in hypertensive patients remains unclear. Different studies reported decreases, increases or no changes in CBF in response to anti-hypertensive medication.^3,4^ Two recent systematic reviews highlighted that many of these studies were small, underpowered, and showed evidence of a significant (moderate-to-high) risk of bias.^3,4^

Here we evaluated the association between systemic arterial blood pressure and cerebral perfusion in a cohort of patients recruited in the Southall and Brent Revisited (SABRE) study, the UK's largest tri-ethnic longitudinal study involving 740 participants^5^ with measurements of cerebral perfusion performed using arterial spin labelling MRI and quantified using a single compartment model without partial volume correction.^6^ The study cohort included white European (41.7%), first South Asian (33.7%) and African Caribbean (23.5%) participants (327 females, 413 males; mean (SD) age 71(7) years). Office blood pressure measurements were obtained on the same day when cerebral perfusion was assessed. The participants’ data were excluded from the analysis if records were incomplete and/or recorded grey matter perfusion values were lower than 20 ml/100 g/min. Data obtained in 690 participants were included in the final analysis. Recorded values of systolic blood pressure and patient history of hypertension were used to stratify the study cohort into 4 groups: i) individuals with normal blood pressure [243 participants; mean systolic blood pressure (SD) 117(9) mmHg; mean (SD) age 70(7) years]; ii) patients with untreated hypertension, not receiving any anti-hypertensive drugs [250 participants; systolic mean 145(12) mmHg; age 72(7) years]; iii) patients with hypertension, controlled on medication with a systolic blood pressure of <130 mmHg [94 participants; systolic mean 117(9) mmHg; age 71(6) years]; and iv) patients with hypertension, prescribed with anti-hypertensive drugs, but remaining uncontrolled on medication with a systolic blood pressure of ≥130 mmHg [103 participants; systolic mean 144(12) mmHg; age 72(6) years].

Accounting for the covariants of age, body mass index (BMI) and sex, a significant negative correlation between systolic arterial blood pressure and cerebral perfusion in both grey (p = 0.01, R^2^ = 0.05) and white matter (p = 0.001, R^2^ = 0.06) was observed across the whole patient cohort (Figure 1(a) to (c)). Subsequent group-wise analysis revealed a difference between participants with normal blood pressure and both unmedicated participants with hypertension (p = 0.02, grey matter; p = 0.002 white matter) and patients with high blood pressure, who were prescribed but remained uncontrolled on medication (p = 0.004, grey matter; p = 0.007; white matter). The mean CBF (SD) in the participants with normal blood pressure was 39(7) mL/100 g/min in the grey matter and 14.5(4) mL/100 g/min in the white matter (n = 243). The lowest values of CBF were recorded in the group of patients with high blood pressure that were uncontrolled on medication 36(6)mL/100 g/min in the grey matter and 13(3) mL/100 g/min in the white matter (n = 103; Figure 1(d) and (e)).

**Figure 1.**
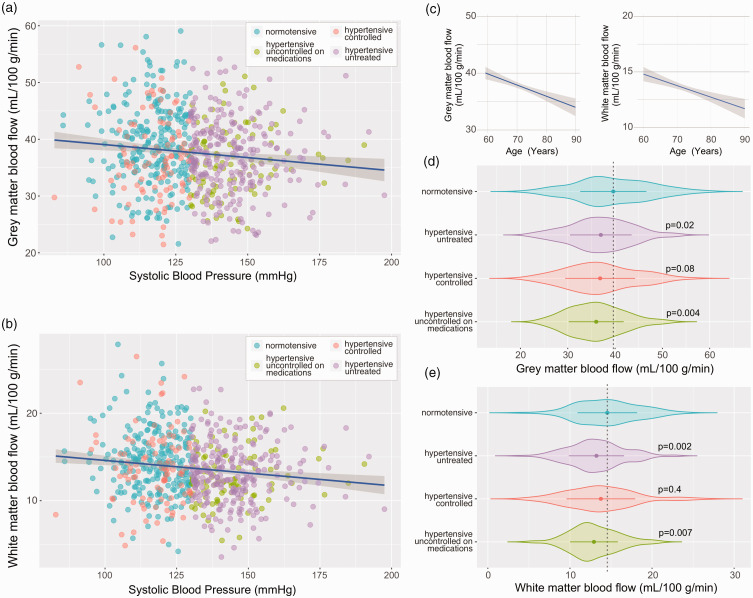
Cerebral perfusion in untreated, controlled, and uncontrolled hypertension. Summary plots illustrating the relationships between grey (a) and white (b) matter perfusion and systolic arterial blood pressure (SBP). Linear fit regression line is shown ± 95% CI. Participant’s medication status groups are indicated: (i) individuals with normal blood pressure, not taking any anti-hypertensive medication with a SBP of <130 mmHg; (ii) patients with untreated hypertension, not taking any anti-hypertensive medication with a SBP ≥130 mmHg; (iii) patients with controlled hypertension, taking anti-hypertensive medication with a SBP of <130 mmHg; and (iv) patients with hypertension, prescribed with anti-hypertensive drugs, but uncontrolled on medication with a SBP of ≥130 mmHg. (c) The relationship between age and cerebral perfusion measured by ASL. Linear fit regression line is shown ± 95% CI and (d,e) summary data illustrating the differences in grey and white matter perfusion between 4 groups of patients. Groups were compared using ANCOVA with age, BMI, and sex as covariants and Tukey’s multiple comparison correction for post-hoc analysis. Violin plots show arithmetic mean (central point)  ± SD with the outline illustrating kernel probability density. Indicated P values were obtained in comparison to the normotensive group data.

These data confirm that high systemic arterial blood pressure is associated with reduced cerebral perfusion. The data do not support the results of a previous study that reported no significant CBF differences between normotensive subjects and individuals with untreated hypertension.^7^ In a recent longitudinal study undertaken in older (mean age 77 years) individuals with hypertension, the use of anti-hypertensive medication was found to be associated with lower grey matter CBF.^8^ In contrast, another recent study involving participants aged 50 years or older (mean age 67.5 years) showed that intensive anti-hypertensive treatment (to achieve systolic blood pressure target of <120 mmHg) was associated with increased, rather than decreased, perfusion in the grey and white matter.^9^ Our analysis suggests that anti-hypertensive treatment has no deleterious effect on cerebral perfusion if systemic arterial blood pressure is controlled on medication and supports the conclusions of the meta-analysis conducted by Van Rijssel and colleagues.^4^ These findings highlight the importance of blood pressure control not only for the purpose of reducing the risk of stroke, cardiovascular disease, heart and renal failure, but also for the benefit of maintaining adequate cerebral blood flow and protecting the brain from harmful effects of chronically reduced perfusion.
